# Unusual Surface Latent Heat Flux Variations and Their Critical Dynamics Revealed before Strong Earthquakes

**DOI:** 10.3390/e24010023

**Published:** 2021-12-23

**Authors:** Soujan Ghosh, Swati Chowdhury, Subrata Kundu, Sudipta Sasmal, Dimitrios Z. Politis, Stelios M. Potirakis, Masashi Hayakawa, Suman Chakraborty, Sandip K. Chakrabarti

**Affiliations:** 1 Indian Centre for Space Physics, 43, Chalantika, Garia Station Road, Kolkata 700084, India; soujanghosh89@gmail.com (S.G.); chowdhuryswati93@gmail.com (S.C.); mcqmld@gmail.com (S.K.); meet2ss25@gmail.com (S.S.); sandipchakrabarti9@gmail.com (S.K.C.); 2 Department of Electrical and Electronics Engineering, Ancient Olive Grove Campus, University of West Attica, 12241 Egaleo, Greece; d.z.politis@uniwa.gr; 3 Hayakawa Institute of Seismo Electromagnetics Co., Ltd., University of Electro-Communications Alliance Center, 521, 1-1-1 Kojma-cho, Chofu, Tokyo 182-0026, Japan; hayakawa@hi-seismo-em.jp; 4 Advanced & Wireless Communications Research Center, University of Electro-Communications, Chofu, Tokyo 182-8585, Japan; 5 Physical Research Laboratory, Navrangpura, Ahmedabad 380009, India; suman.chakrabarty37@gmail.com

**Keywords:** earthquake, thermal anomaly, surface latent heat, natural time analysis, criticality, LAIC

## Abstract

We focus on the possible thermal channel of the well-known Lithosphere–Atmosphere–Ionosphere Coupling (LAIC) mechanism to identify the behavior of thermal anomalies during and prior to strong seismic events. For this, we investigate the variation of Surface Latent Heat Flux (SLHF) as resulting from satellite observables. We demonstrate a spatio-temporal variation in the SLHF before and after a set of strong seismic events occurred in Kathmandu, Nepal, and Kumamoto, Japan, having magnitudes of 7.8, 7.3, and 7.0, respectively. Before the studied earthquake cases, significant enhancements in the SLHF were identified near the epicenters. Additionally, in order to check whether critical dynamics, as the signature of a complex phenomenon such as earthquake preparation, are reflected in the SLHF data, we performed a criticality analysis using the natural time analysis method. The approach to criticality was detected within one week before each mainshock.

## 1. Introduction

Earthquake prediction is one of the most challenging targets for scientists for more than a decade [1]. Various kinds of precursory phenomena have been observed and reported before large devastating earthquakes (see Molchanov et al. [2], Ouzounov et al. [3], Pulinets and Boyarchuk [4]), and those phenomena are categorized into three different types, namely, electromagnetic, acoustic, and thermal channels [2,5]. We have published several reports on electromagnetic and acoustic anomalies connected to earthquakes [6,7,8]. In this paper, we focus mainly on the thermal phenomenon which belongs to the thermal channel and its related anomaly. The study of surface thermal anomalies will be a fundamental basis for the elucidation of the lithosphere–atmosphere–ionosphere coupling (LAIC) process as the initial agent of the LAIC mechanism. Before earthquakes, rock pressure mainly triggers a temperature increase on the land’s surface. Thermal infrared waves are emitted due to the stress accumulation arising out of this rock pressure. Ouzounov et al. [9] and Surkov et al. [10] found that the enhancement in thermal activities is actually due to these waves.

In the 1980s, researchers in Central Asia found a short-lived but significant signature of thermal anomalies before earthquakes from satellite images [11]. From that point forward, numerous researchers started to study these sorts of thermal anomalies from various satellite data as an antecedent to earthquakes in China, Japan, India, Iran, and Algeria [9,12,13,14,15,16]. Researchers mainly concentrate on satellite data because satellite remote sensing has some advantages over traditional approaches of ground-based seismic hazard monitoring. The first advantage of satellite data is the high spatio-temporal resolution obtained from advanced satellite technology. Satellite Thermal Infrared data (TIR) are also considered to be one of the most reliable data sources as they exhibit continuity, stability, and easy accessibility of the data. Due to the high-resolution and global coverage of the continuous data, one can monitor the fast-changing and large-scale phenomena related to seismic activity with the satellite observation. In addition, it has been found, from different examinations, that satellite TIR information can uncover large-scale linear structures in addition to short term (i.e., from days to weeks) variations of thermal abnormalities over tectonic plate outskirts and active faults [15,17,18,19]. Several scientists have studied various cases of thermal anomalies from satellite data and have found a similar type of results in active seismic areas before earthquakes with moderate or strong magnitude.

To contemplate the thermal anomaly before and also at the hour of large earthquakes, we choose the meteorological parameter, Surface Latent Heat Flux (SLHF). Surface Latent Heat Flux (SLHF) is the key component of the Earth’s radiation budget. It is defined as the heat released during the phase change of matter on the earth’s surface. Heat energy is transported from the Earth’s surface to the atmosphere through the evaporation process. This evaporation process compensates for the energy loss due to the radiation processes [20]. Moreover, the evaporation rate is higher on the ocean surface compared to the land surface. Similarly, the SLHF is also higher in the ocean than on the land. Before large earthquakes, a thermal infrared emission is observed near the epicentre [9]. This emission of thermal infrared occurs due to the accumulation of stress in the fault regions. Due to the thermal infrared emission, the rate of exchange of energy from the surface to the atmosphere increases. As a result, the SLHF also increases before strong earthquakes [21]. SLHF is also dependent on various atmospheric parameters such as air temperature and relative humidity. According to the LAIC mechanism, radon is the primary source of ionization in the surface area. The newly formed ions are attached to water molecules present in the air. Hence, the trend of air temperature and relative humidity varies from normal during the preparation time of the earthquakes near the epicenter. In addition, there is a growth in the ion clusters due to hydration. These cluster formations change the air conductivity and generate the anomalous electric field which, finally, changes the ionospheric potential. On the other hand, change in air temperature and relative humidity create vertical thermal convection and an air pressure gradient. Vertical thermal convection also modifies the atmospheric electric field and creates convection currents. The gradient in air pressure generates atmospheric gravity waves. All these changes in the convection current and atmospheric electric field create ionospheric perturbations. So, the change in the SLHF is a key parameter in the LAIC mechanism.

For coastal earthquakes, Dey and Singh [21] have observed an anomalous increase in the SLHF 20 days before the earthquake. So, the SLHF anomaly can be considered as a possible precursor in giving early data about a looming seaside seismic event. Cervone et al. [22] have developed a wavelet analysis approach to distinguish maximum peaks related to an approaching earthquake and demonstrate atmospheric disturbances. Pulinets et al. [23] have suggested that the abnormal increase in the SLHF a few days prior to the earthquakes in coastal areas is actually due to the increase of surface temperature in a seismically prone area. For various reasons, the SLHF abnormality is fundamentally more “fragile” on the land surface than on the ocean surface. The main reasons that affect the surface latent heat anomaly are air humidity, surface temperature, and wind speed. Since there is a significant difference between sea and land surface variations of these parameters, the anomalous behavior of the SLHF is also affected. Studies of Qin et al. [24] showed that a strange change in the SLHF occurred almost 10 days before the inland Pu’er earthquake (geographic coordinates: 23.8092${}^{\circ }$ N, 101.15${}^{\circ }$ E) on 2 June 2007. Though Zhang et al. [25] found some opposite results which show that no huge irregular variation in the SLHF exists preceding around 10 cases of marine or coastal earthquakes. Hence, it is clear that in order to establish the connection between the SLHF abnormality and an earthquake’s occurrence, information exactness and boundary settings have a noteworthy role.

The significant change in the SLHF irregularity before large earthquakes is strongly identified with the underground fluid motion and the communication among the underground, the surface, and the air [26]. Mansouri et al. [27] indicated that water vapor reactions to seismic preparation stages follow a seismic-activated chain, which is the fundamental mechanism behind air ionization, SLHF intensification, and water vapor buildup, and this can likewise bring about the development of precipitation activity. Several authors have found that SLHF enhancement before earthquake events are keenly related to soil moisture [21,22]. This soil moisture is responsible for air ionization and SLHF oddities in the LAIC process. With a rise in soil moisture, there is a noteworthy change in the expansion of water condensation on recently formed ions [28].

A few examinations propose that the thermal inconsistencies in epicentral areas and the adjunct regions before large earthquakes are identified with the expansion in both air and soil temperature at shallow layers. Temperature inconsistency demonstrated by MODIS data and from other meteorological stations is found to be near to the epicenter [29]. The seismic distortion and fault evolution under seismic pressure changes are the main reason behind the abnormality of temperature variation [30]. Thus, temperature anomaly is a more powerful tool in identifying thermal abnormalities than Top of Atmosphere (TOA) brightness temperature or Outgoing Long-wave Radiation (OLR), since it is less influenced by the atmosphere, which permits us to obtain a higher signal-to-noise ratio [31,32]. Tramutoli et al. [33] and Tronin et al. [15] have found temperature increments up to 5 ${}^{\circ }$C before seismic events in Italy, Japan, and China. For the Indian sub-continent, similar types of results were reported by the study of Ouzounov et al. [9]. Their study using MODIS data near the active tectonic fault of the Gujarat earthquake in India (M = 7.7, 26 January 2001) revealed an abnormality in the land surface temperature and a bringing down of the sea surface temperature. Tramutoli et al. [18] found some satisfactory results for the Izmit earthquake in Turkey (M = 7.4, 19 August 1999) by improving their 2001 technique for the analysis of thermal anomalies before large seismic events. Jing et al. [34] reported convincing evidence of the coupling between the land, atmosphere, and meteorological parameters associated with the 2015 Nepal earthquake. In that paper, they simultaneously used the microwave brightness temperature, the surface air temperature, the soil moisture, the surface latent heat, andthe carbon monoxide profile as a multi-parametric examination of the precursory mechanism associated with Nepal earthquakes. Their study concluded that the thermal variations in the sub-surface show anomalous behavior two months prior to the mainshock, where the other anomalies appeared two weeks before.

**Figure 1:**
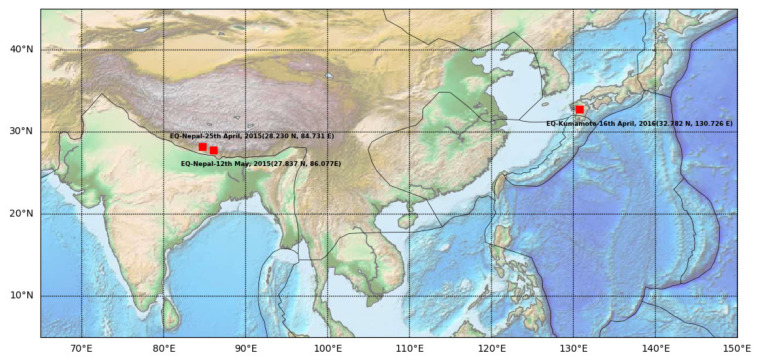
The locations of the epicenter of the earthquakes.

In our study, we have chosen the Nepal earthquakes of 2015 and the Kumamoto earthquake of 2016. On 25 April 2015, at UTC 06:11:25, a strong (M = 7.8) earthquake occurred in Nepal. The epicenter was at Barpak east of the Gorkha district. After this earthquake, a chain of aftershocks hit Nepal, and again on 12 May 2015, another earthquake (M = 7.3) hit the southeast of Kodari. In 2016, a series of earthquakes hit the coast of Japan, including a magnitude M = 7.0 mainshock which struck at16:25 UTC on 15 April underneath Kumamoto City of Kumamoto Prefecture in Kyushu Region, Japan, and a foreshock of M = 6.2 at 12:26 UTC on 14 April 2016 at a depth of about 11 km. In Figure 1, we present the location of the epicenter of the earthquakes with the main tectonic and geological features. To investigate the possible pre- and co-seismic SLHF variations, we use the conventional spatio-temporal study of the increasing SLHF by removing the background variation of non-seismic conditions. We also study the SLHF anomaly variation for these earthquakes considering the latitude and longitude of the epicenters as the center point and present the latitudinal and longitudinal variation in the SLHF for that time period. Moreover, we study the SLHF time series at the closest possible to each earthquake’s epicenter location by means of the natural time analysis [35], a powerful time series analysis method that can unveil the approach to criticality. Our results indicate that the analyzed time series present critical dynamics within one week before each mainshock. Finally, the obtained results are discussed within the frame of LAIC.

## 2. Data and Methodologies

Advanced satellite technology provides high-resolution spatio-temporal global remote sensing data. Satellite Thermal infrared (TIR) data are significantly solid information source that displays the progression, dependability, and availability. This information provides the scope of investigation of large scale and rapid changing thermal irregularities associated with seismic events. Crustal deformation and related thermal information of the Earth’s interior can occur very rapidly on a perceptible scale using satellite TIR abnormality. In addition, large scale linear structure and fast-changing impermanent (i.e., from days to weeks) changes in thermal abnormalities in the outskirts of tectonic plate boundary and active faults can be uncovered by satellite TIR data. We take National Oceanic and Atmospheric Administration (NOAA) reanalysis data for Nepal and Kumamoto, Japan, earthquakes and analyze those data to study the thermal anomaly. NOAA reanalysis data are taken from https://www.esrl.noaa.gov (accessed on 23 September 2018). NOAA Reanalysis data were created with the collaboration of other organizations and cover data from 1948 to the present day. These reanalysis datasets are created based on surface, upper-air balloon, aircraft, and satellite observations. These datasets are available in three formats: four times daily, daily, and monthly. These datasets are divided into four different classes A, B, C, and D. The A class parameters are directly obtained from observations, whereas the B class parameters are also obtained directly with strong influence from model data. C class parameters are obtained from the model. D class parameters are not dependent on model values. Now, the SLHF parameter belongs to the C class [36]. Zonal wind, Meridional wind, and geopotential height are some class A parameters, whereas surface temperature, surface pressure, and relative humidity are class B parameters. Ice concentration is an example of D class parameter. As the SLHF parameter is not assimilated but directly derived from the model, we have not addressed the assimilation methodology in this paper. In our case studies, we choose surface level daily data. To identify the thermal anomaly related to earthquakes, we need to remove diurnal, seasonal variation, and other climatological and meteorological effects. To remove these effects, we prepare background data using the seismically quiet period for the same grid area. To compute the background data, we use the following equation:

$$G_{bac}\left(x,y,t\right)=\frac{1}{N}\sum_{i=1}^{N}G_{i}\left(x,y,t\right).$$

For both, the studied cases the value of *N* is 3. In the case of the Nepal earthquake, we prepared the background using the data for the years 2016, 2017, and 2018, which were seismically quiet. For Japan, we chose similar variations by using the data for 2017, 2018, and 2019 to prepare the background variation. Now, in order to extract the actual seismogenic variation, we subtract that background variation from the seismically active time period as follows:

$$Anomaly\left(x,y,t\right)=[G\left(x,y,t\right)-G_{bac}\left(x,y,t\right)],$$

where G(x,y,t) is the data obtained for the seismically active time period. For the Nepal earthquake, we used the latitude range 22${}^{\circ }$ N to 34${}^{\circ }$ N and the longitude range of 80${}^{\circ }$ E to 92${}^{\circ }$ E for the spatio-temporal variation. We compute the anomaly in the SLHF parameter and overlay the world map over the results to recognize the spatio-temporal variation regarding the epicenter and earthquake fault line. For the Kumamoto earthquake, we use latitude range 25${}^{\circ }$ N to 39${}^{\circ }$ N and longitude range 121${}^{\circ }$ E to 136${}^{\circ }$ E. We also overlay the world map over the results to recognize the spatio-temporal variation regarding the epicenter and earthquake fault line. Apart from the spatio-temporal variation, in order to recognize the spatial trend of the SLHF and the possible impact of the aftershocks, we perform a separate analysis. The SLHF has been studied as a function of the day number and latitude of the earthquake epicenter with fixed longitude and vice versa. For the SLHF parameter, the latitude and longitude resolution of the raw data are taken as 1.9${}^{\circ }$ and 1.875${}^{\circ }$, respectively. Interpolated data files are created with a 0.09${}^{\circ }$× 0.09${}^{\circ }$ binning grid that displays a smooth spatio-temporal variation. We interpolate the gridded values of SLHF to 1${}^{\circ }$ interval for both latitudinal and longitudinal profiles for the total time period of interest. We obtain such temporal variations in latitudinal and longitudinal profiles just to emphasize the expansion of the above-mentioned anomaly around the earthquake epicenter.

Finally, in order to apply the natural time analysis (see Section 3) and check for any approach of SLHF to criticality, we need an SLHF time series at a specific geographical point. Since for the spatio-temporal analysis the spatial resolution grid has been taken as 1${}^{\circ }$× 1${}^{\circ }$, we examine the nearest grid point to each mainshock epicenter (see Table 1). At that particular point, we find the SLHF variation along with the deviation of SLHF computed from seismically quiet years, from which we produce the daily-valued time series $\Delta SLHF\left(t\right)=SLHF_{nearest\;grid\;point}\left(t\right)-SLHF_{nearest\;grid\;point\;quiet}\left(t\right)$. We clarify that the considered nearest grid points are within the critical radius, which, for an EQ of magnitude M7, is 200 km [37].

## 3. Natural Time (NT) Analysis Method

NT time series analysis method has initially been applied to the ultra-low frequency (≤1 Hz) seismic electric signals (SES) and foreshock seismicity [38,39,40] and has been shown to be optimal for enhancing the signals in the time-frequency space [41]. Subsequently, NT analysis has been applied to various, real-world and simulated, complex systems time series to reveal their approach to criticality [35], including several seismo-electromagnetic signals [42], as well as air ion density anomalies [43] and global navigation satellite system surface deformation [44] possibly related to earthquakes and the LAIC phenomenon. In the following, we briefly present the key notions of this method. The interested reader is referred to Potirakis et al. [42] for a detailed presentation of the practical application of NT analysis to various time series, including daily-valued ones as the here investigated ΔSLHF, as well as to Varotsos et al. [35], for its full theoretical presentation.

Initially, for a number of *N* events, we determine the NT of the occurrence of the k-th event as $\chi_{k}=k/N$, i.e., the conventional time information is removed, and only the order of occurrence is retained, normalized in the range [0, 1]. Next, we determine the “energy” of each event in NT, which is symbolized as $Q_{k}$ for the k-th event. At this point we have to mention that $Q_{k}$ corresponds to different kinds of quantities, depending on the time series under analysis. For example, in the case of seismic events, $Q_{k}$ is the seismic energy released (seismic moment), while for the dichotomous SES signals, $Q_{k}$ corresponds to the SES pulse duration [40]. However, in the case of the fracto-electromagnetic emission signals in the MHz band, which are non-dichotomous signals, $Q_{k}$ denotes the energy of each event by using consecutive amplitude values above a noise threshold as described in Potirakis et al. [45].

Then, we study the evolution of the pair of $\left(\chi_{k},Q_{k}\right)$. The approach of a dynamical system to criticality is identified by means of the variance of NT, $\kappa_{1}=\langle \chi^{2}\rangle -{\langle \chi \rangle }^{2}=\sum_{k=1}^{N}p_{k}\chi_{k}^{2}-{\left(\sum_{k=1}^{N}p_{k}\chi_{k}\right)}^{2}$, where $p_{k}=\frac{Q_{k}}{\sum_{n=1}^{N}Q_{n}}$ is the normalized energy released during the k-th event. Moreover, the entropy $S_{nt}$ in NT is defined as $S_{nt}=\sum_{k=1}^{N}p_{k}\chi_{k}ln\chi_{k}-\left(\sum_{k=1}^{N}p_{k}\chi_{k}\right)ln\left(\sum_{k=1}^{N}p_{k}\chi_{k}\right)$, which corresponds to the value for q =1 of the derivative of the fluctuation function with respect to q, fl(q) (while $\kappa_{1}$ corresponds to fl(2)) [35,46]. The entropy in NT is a dynamic entropy, depending on the order of the events [46]. Moreover, $S_{nt-}$, the entropy under time reversal ($Tp_{m}=p_{N-m+1}$), is also studied [46].

In many studied dynamical systems, it has been found that the value of $\kappa_{1}$ is a measure to quantify the extent of the organization of the system at the onset of the critical stage [35]. The criticality is reached when (i) $\kappa_{1}$ takes the value $\kappa_{1}=0.07$, and (ii) at the same time, both the entropy in NT and the entropy under time reversal satisfy the condition $S_{nt},S_{nt-}<S_{u}=\left(ln2/2\right)-1/4$ [35,47], where $S_{u}$ is the entropy of the uniform distribution in NT [35,46].

In the special case of NT analysis of foreshock seismicity [38,40,41,46,48], we study the evolution of the quantities $\kappa_{1},S_{nt},S_{nt-}$, and 〈D〉 with time, where 〈D〉 is the “average” distance between the normalized power spectra $\prod \left(\tilde{\omega }\right)=|\sum_{k=1}^{N}p_{k}exp\left(j\tilde{\omega }\chi_{k}\right){|}^{2}$ ($\tilde{\omega }$ stands for the angular frequency in NT) of the evolving seismicity and the theoretical estimation of $\prod (\tilde{\omega })$ for $\kappa_{1}=0.07$, $\prod_{critical}\left(\tilde{\omega }\right)\approx 1-\kappa_{1}{\tilde{\omega }}^{2}$. Moreover, an “event” for the NT analysis of seismicity is considered to be any data point (earthquake) of the original seismicity time series that surpasses a magnitude threshold, $M_{Thres}$.

The analysis starts with an appropriate low threshold and taking into account only an adequate number of events, first in the order of occurrence. Next, the subsequent events, in their original order, are one-by-one taken into account. For each additional event that is taken into account, the quantity $\chi_{k}$ is rescaled within the interval (0,1), while the normalized energy $p_{k}$ and all $\kappa_{1},S_{nt},S_{nt-}$, and 〈D〉 are re-calculated. This way, a temporal evolution of these quantities is attained, taking into account the current event and all preceding events. The described procedure is repeated for several, increasing values of $M_{Thres}$ for each studied geographic area, and everything is repeated for different overlapping areas.

The seismicity is considered to be in a true critical state, a “true coincidence” is achieved, as soon as (i) $\kappa_{1}$ takes the value $\kappa_{1}=0.07$, (ii) at the same time both the entropy in NT and the entropy under time reversal satisfy the condition $S_{nt},S_{nt-}<S_{u}$, and three additional conditions are satisfied: (iii) The “average” distance 〈D〉 should be smaller than $10^{-2}$, i.e., $\langle D\rangle =\langle |\prod \left(\tilde{\omega }\right)-\prod_{critical}\left(\tilde{\omega }\right)|\rangle <10^{-2}$ (this is a practical criterion for signaling the achievement of spectral coincidence) [35]; (iv) the parameter $\kappa_{1}$ should approach the value $\kappa_{1}=0.07$ “by descending from above”, i.e., before the main event the parameter should gradually decrease until it reaches the critical value 0.070 (this rule was found empirically) [35,38]; (v) the above-mentioned conditions (i)–(iv) should continue to be satisfied even if the considered $M_{Thres}$ or the area within which the seismicity is studied are changed (within reasonable limits).

The use of the magnitude threshold excepts some of the weaker earthquake events (those events that their magnitude is <$M_{Thres}$) from the NT analysis. However, the usage of the magnitude threshold is valid for the reason that some recorded magnitudes (lower than a threshold) are not considered reliable due to the seismographic network. On the other hand, the application of various $M_{Thres}$ values is useful in determining the time range within which criticality is reached. This is because, in some cases, it is found that more than one time-points may satisfy the rest of the NT critical state conditions (i)–(iv), and criterion (v) is the one that finally reveals the true time of criticality. For the application of NT analysis to ΔSLHF data, we follow the paradigm of the NT analysis of foreshock seismicity by using the absolute value of their daily values to define the “energy” and the necessary threshold values.

## 4. Results and Discussion

### 4.1. SLHF Observational Results

The representation of the variation in the SLHF has been performed in two ways. First, we proceed with the spatio-temporal variation which gives the direct evidence of the increase in the SLHF.

**Figure 2:**
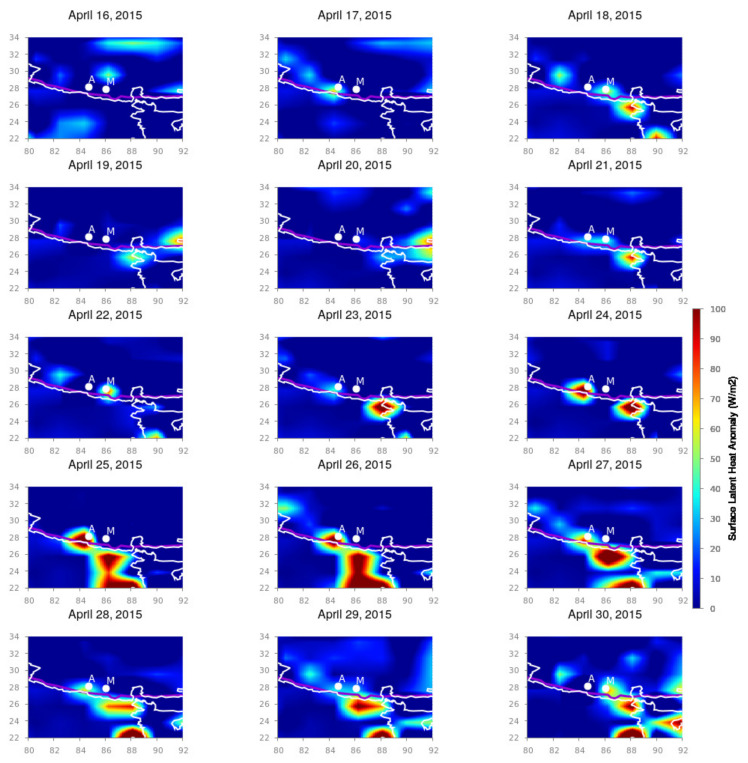
SLHF variation for 16 April to 30 April 2015, around the epicenter of Nepal earthquakes. Along the X and Y axes, we have presented the geographic longitude and latitude, respectively. The white outlines are the country border, and the white dots are the epicenters of the earthquakes. `A’ and `M’ represent the epicenters of the 25 April and 12 May earthquakes, respectively.

**Figure 3:**
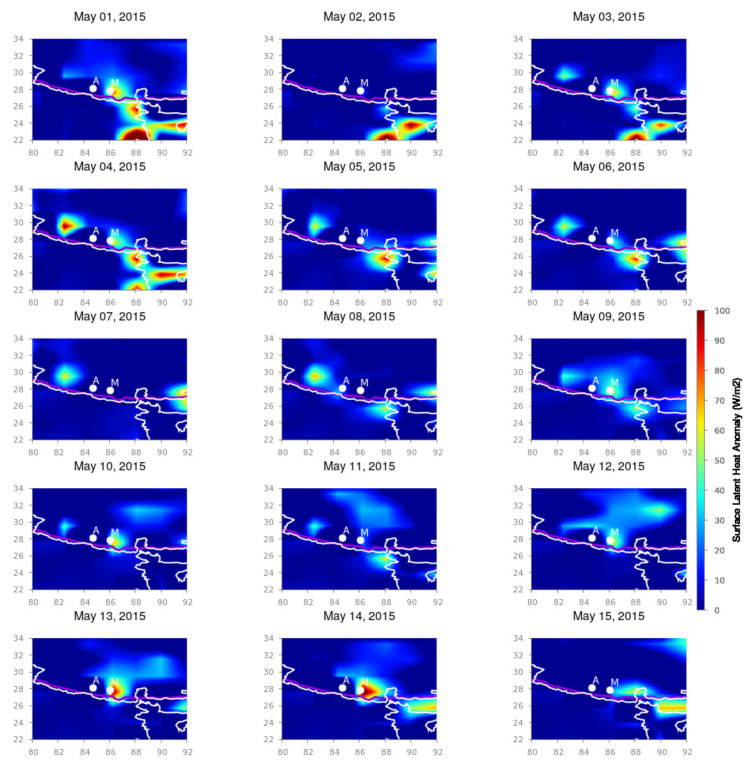
SLHF anomaly variation during 1–15 May 2015. Figure format follows the format of Figure 2.

**Figure 4:**
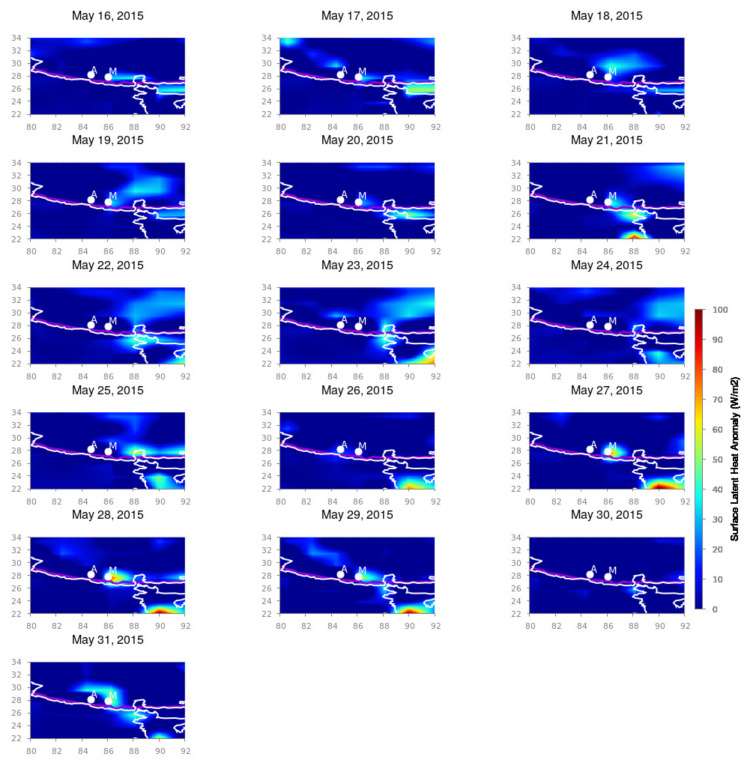
SLHF anomaly variation during 16–31 May 2015. Figure format follows the format of Figure 2.

For the Nepal earthquakes, we present the surface latent heat anomaly variation for the months of April and May 2015 from longitudinal and latitudinal spans from 80${}^{\circ }$ E to 92${}^{\circ }$ E and 22${}^{\circ }$ N to 34${}^{\circ }$ N, respectively, which cover the total Nepal region with its surrounding areas. Figure 2, Figure 3 and Figure 4 depict the spatio-temporal variation in the SLHF after subtracting the background variation during seismically quiet periods. The white outline is the country border and the white dots are the epicenters of the 25 April and 12 May Nepal earthquake, respectively. `A’ and `M’ indicate the locations of the epicenters of the April and May earthquakes, respectively.

As shown in Figure 2 and Figure 3, sudden intensification happened in the SLHF on 21 April 2015. After that, a smaller patch of intensive SLHF develops around the seismic fault lines, and from 24 April, the anomaly becomes more prominent and gradually spreads around the epicenter. The first mainshock happened on 25 April, and this intensification can be associated with the possible pre-seismic excitation of the SLHF anomaly. It continues for the entirety of May, with more scattered way up to the first week of May. During this time, a series of moderate aftershocks took place, which remained until 5 May 2015. We anticipate that the post-earthquake anomaly is due to the effects of series of a such an aftershock. It is also evident that the SLHF has a continuous spreading around the epicenter with less intensity than that for the mainshock. For the 12 May earthquake, the behavior is rather different. During 9–12 May, the SLHF is highly prominent exactly over the epicenter but with a fainter magnitude than that for the 25 April earthquake. It obtains more energy after the mainshock, and during 13–14 May, the intensification is rather high, but the spatial spread is lower as compared to the 25 April earthquake. The 12 May earthquake is also followed by a series of aftershocks but with a smaller magnitude. For 16 May, the enhancement of the SLHF stops, and no more anomaly can be detected through the spatio-temporal variation, as seen in Figure 4. This can be attributed to the termination of further seismic activities.

**Figure 5:**
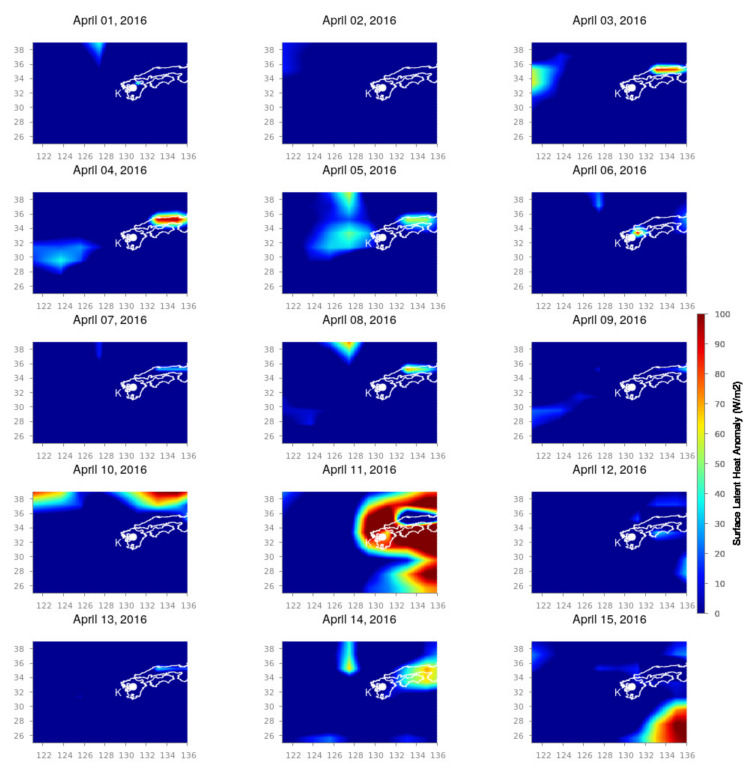
SLHF variation during 1 to 15 April 2016 for the Kumamoto earthquake. The white outlines are the country border, and `K’ represents the epicenter of the Kumamoto earthquake.

**Figure 6:**
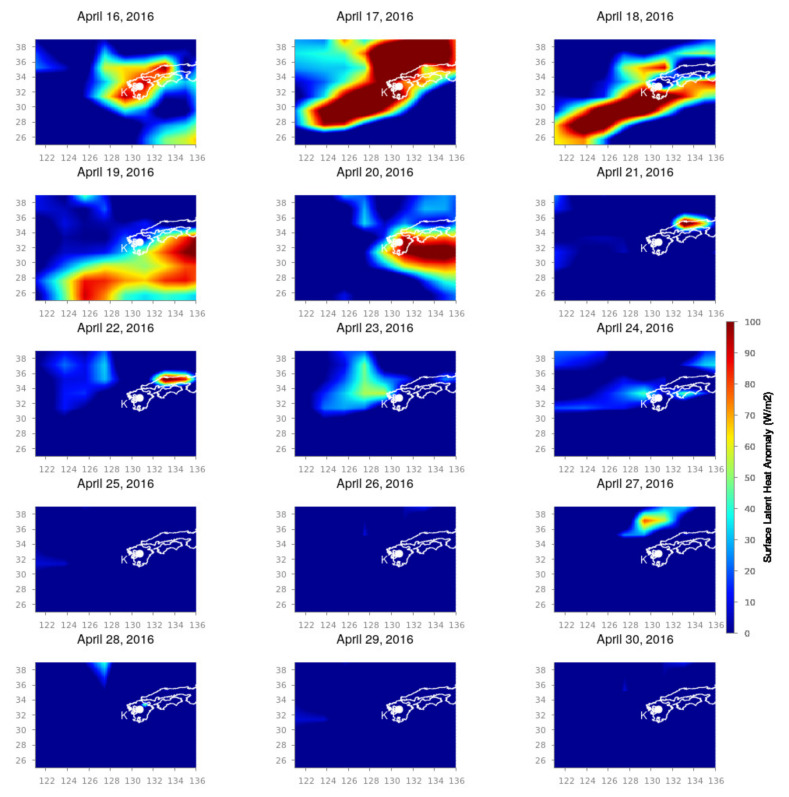
SLHF variation during 16 to 30 April 2016. Figure format follows the format of Figure 5.

The Kumamoto earthquake shows a slightly different variation in comparison to the Nepal earthquake. The SLHF variation for the Kumamoto earthquake is shown in Figure 5 (1 to 15 April 2015) and Figure 6 (16 to 30 April 2015). We set the spatial span in longitude 120${}^{\circ }$ E to 136${}^{\circ }$ E and latitude from 25${}^{\circ }$ N to 39${}^{\circ }$ N to cover the corresponding earthquake region and its surrounding areas. `K’ is the location of the epicenter. From Figure 5, it is evident that there is a sudden enhancement in SLHF on 10 April 2016 and quickly spreads out over the epicenter on 11 April 2016. Here, we point out that these dates are the period with the most enhanced ionospheric perturbation [49,50]. After this date, it diminishes. This can be explained by the foreshock on 14 April 2016. From 15 April again, a strong patch of SLHF is generated from the south, and on 16 April, it covers the entire epicenter and its surroundings (Figure 6). The intensification becomes enhanced for the coming few days (17 to 20 April 2016), which indicates the post effects of earthquakes as generated by the series of strong aftershocks. From 21 April, the intensification fades away, and it reaches the normal value.

**Figure 7:**
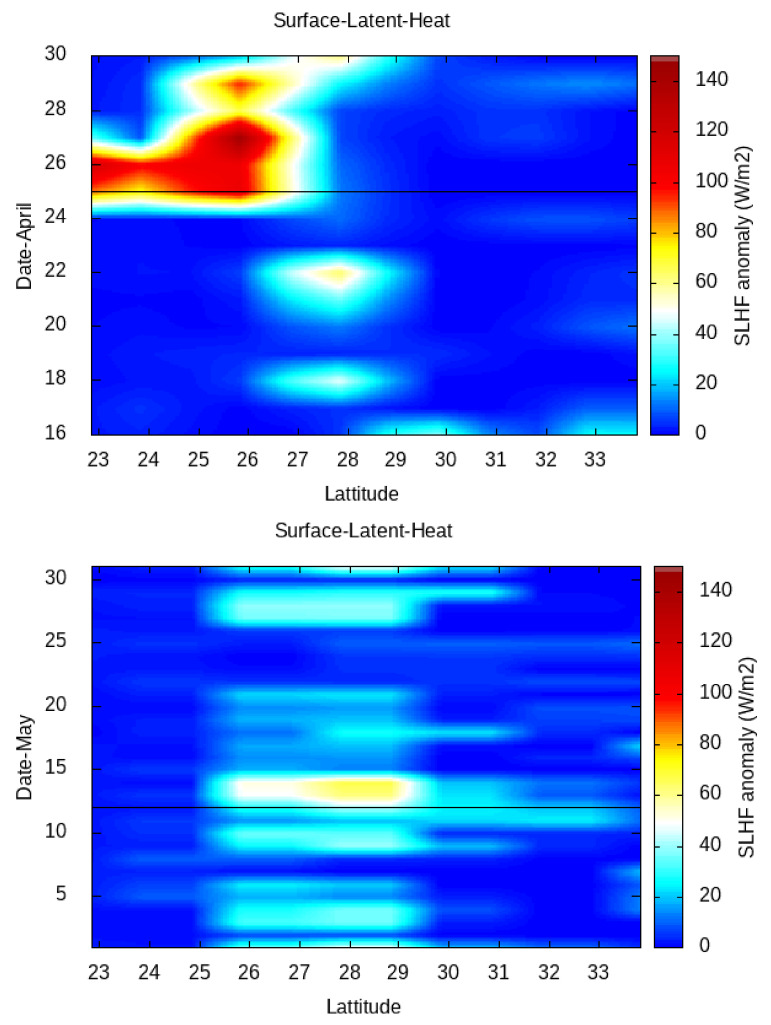
Latitudinal variation in SLHF for the April (**top**) and May (**bottom**) 2015 Nepal earthquakes. The horizontal line indicates the corresponding earthquake date.

**Figure 8:**
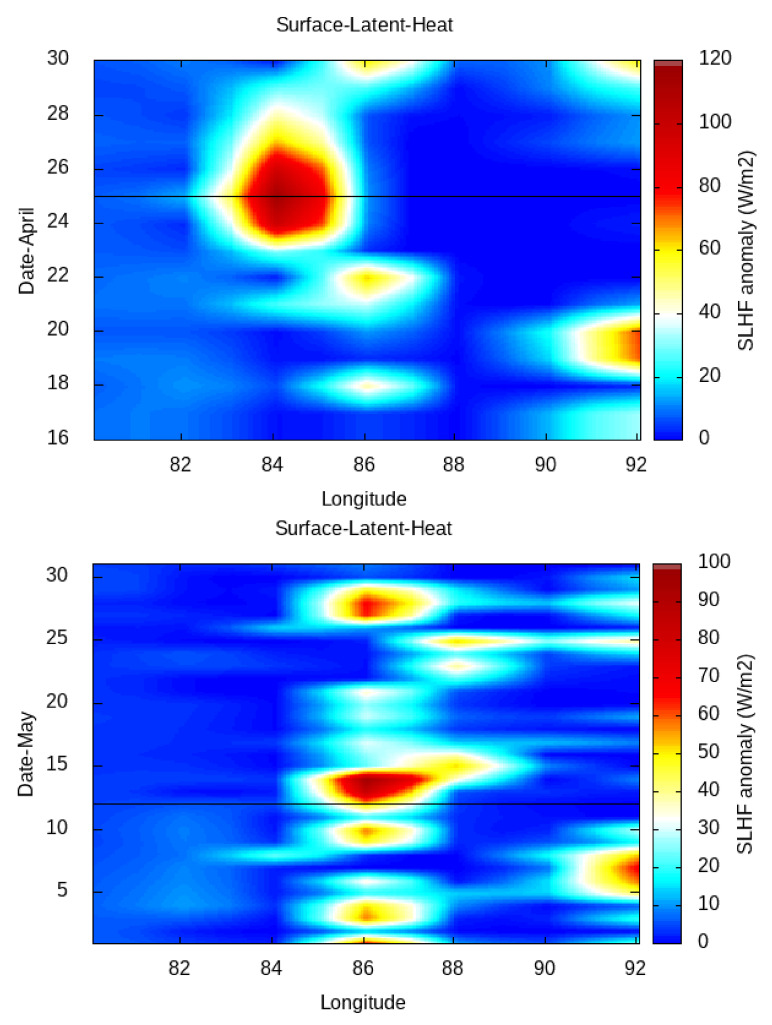
Longitudinal variation in SLHF for the April (**top**) and May (**bottom**) 2015 Nepal earthquakes. The horizontal line indicates the corresponding earthquake date.

**Figure 9:**
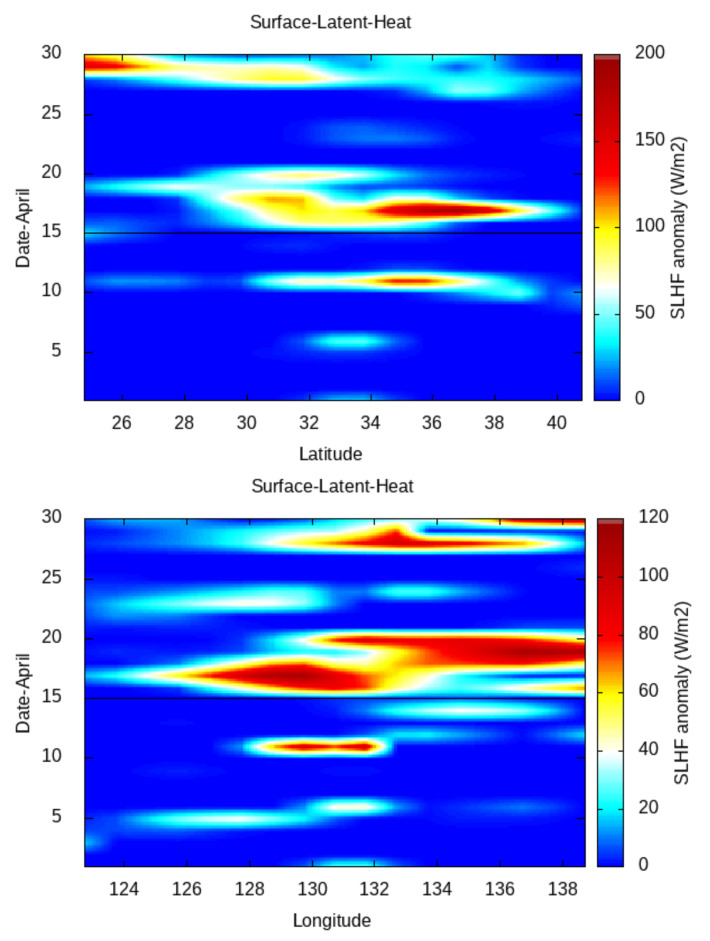
Latitudinal (**top**) and longitudinal (**bottom**) variation of surface latent heat flux for April 2016. Horizontal line indicates the Kumamoto maishock date.

Then, in order to study the latitudinal/longitudinal variation in the SLHF with a 1${}^{\circ }$ spatial interval from the gridded data, we fix the longitude/latitude of the epicenter of the 12 May earthquake as a center grid point (Figure 7 and Figure 8, respectively). As the spatial distance between the April and May earthquakes are not so far, we use a single fixed point for both earthquakes. For Kumamoto, we do the same for the main shock of 15 April 2016 (Figure 9).

The latitudinal/longitudinal variation gives a much better identification of the spreading of the SLHF over the earthquake epicenters. The latitudinal variation in the SLHF anomaly for April and May show a clear patch around the latitude of earthquake epicenters. The maximum intensification happens just around 25 April, and it spreads over the south of the epicenter. After the 25 April earthquake, due to a series of aftershocks, the overall energy budget becomes increased and continues. A similar enhancement with less intensification happens around the 12 May earthquake, and here, the direction of the enhancement is partly in the north direction. As concerned with the longitudinal spread, the variation is rather compact with less spread in comparison with a latitudinal variation. For the 25 April earthquake, a similar enhancement is seen to be highly concentrated over the epicenter. After that, the enhancement with less intensity continues, but on the contrary to the latitudinal case, the intensification due to the aftershocks becomes concentrated around the epicenter of the 12 May earthquake. A similar enhancement happens around 10 to 15 May, explicitly over the earthquake epicenter of 12 May.

In comparison to the Nepal earthquakes, the spatial spread is rather different from that for the Kumamoto case, as seen in Figure 9. The latitudinal (top) and longitudinal (bottom) variation in the SLHF shows a clear abnormality around the epicenter. The enhancement in the SLHF occurs on 10 April (pre-seismic) and during 15–20 April. Here, the intensification spreads over both the north–south and east–west directions over the epicenter.

In Figure 7, Figure 8 and Figure 9, we present the latitudinal and longitudinal variations in the SLHF anomaly, considering the epicenter of the earthquake as the midpoint. We present these Figures to identify the propagation of the SLHF anomaly in the region. In this context, we also need to clarify that while computing and identifying the propagation of the SLHF anomaly, we considered the fixed latitude and longitude of the epicenter as the mid-point. For this consideration, latitudinal variation is not reflecting any longitudinal abnormality other than the epicenter, which is actually present in the spatio-temporal plots. A similar incident occurred for the longitudinal variation also. In those three Figures, we mainly focused on the latitudinal/longitudinal extent of the SLHF anomaly and its propagation. Previously, we have studied the electromagnetic channel of the LAIC mechanism, where we have found that the anomalous behavior of various parameters occurred both pre- and co-seismically [8]. In that particular paper, we found that the maximum shift in VLF sunrise terminator time is observed pre-seismic for the 2015 Nepal EQ but co-seismic for the 2011 Honshu EQ though the magnitude of the 2011 Honshu EQ is much higher than the 2015 Nepal EQ. This result indicates that irrespective of the magnitude, location, geographic condition, and propagation, the path plays a major role in the identification of the precursory phenomena. Here, we also found a similar type of pre- and co-seismic variation for the SLHF parameter.

**Figure 10:**
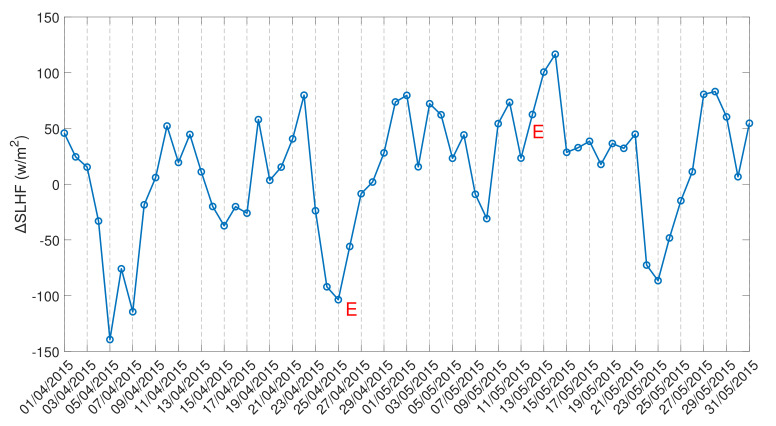
Variation of SLHF at the nearest grid point (ΔSLHF(t)) for Nepal earthquakes. The dates of the mainshocks are indicated by `E’.

**Figure 11:**
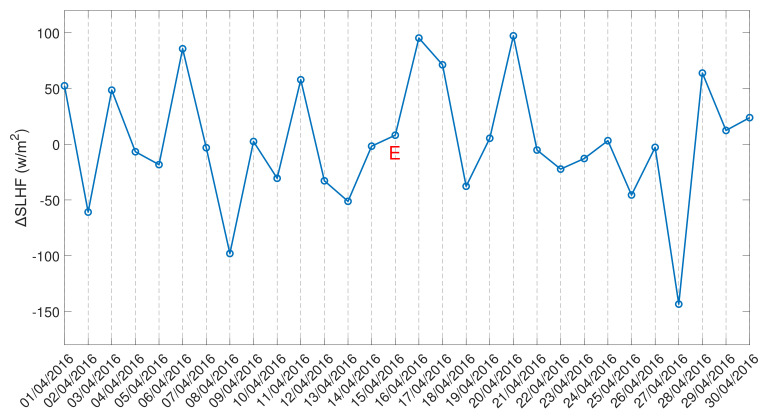
Variation of SLHF at the nearest grid point (ΔSLHF(t)) for Kumamoto earthquake. The date of the mainshock is indicated by `E’.

The spatio-temporal anomaly revealed that SLHF anomaly is presented near the epicenter and propagated towards the epicenter but has also indicated that the nearest grid point may not be in the path of the SLHF anomaly for the entire time periods, which results in the low values of the SLHF variation in Figure 10 and Figure 11. For this reason, ΔSLHF(t) appears random in Figure 10 and Figure 11. We present these two Figures just to show the variation in the ΔSLHF(t) before computing the natural time analysis quantities to identify any criticality before the studied earthquakes and not to draw direct conclusions.

### 4.2. NT Analysis Results

In this first attempt to apply the NT analysis to the SLHF, we use ΔSLHF(t) and the daily-valued time series of the variation in the SLHF at the nearest grid point as defined in Section 2. Figure 10 and Figure 11 present the variation in ΔSLHF for the 2015 Nepal and the 2016 Kumamoto earthquakes, respectively. In order to define positive valued events necessary for the application of NT analysis, we also applied absolute value as the final stage of the original data preprocessing. As already mentioned in Section 3, the application of NT analysis to the ΔSLHF time series follows the paradigm of the NT analysis of foreshock seismicity. That is, we applied the NT analysis procedure described in Section 3 for the foreshock seismicity by using the time series of the absolute value of ΔSLHF, ∣ΔSLHF∣, to define the “energy” $Q_{k}$ and the necessary threshold values ∣ΔSLHF∣${}_{thres}$. Specifically, for each earthquake case (Nepal, Kumamoto), we consider all daily values of ∣ΔSLHF∣ that are higher than a certain threshold ∣ΔSLHF∣${}_{thres}$ as “events” to be taken into account during the NT analysis. The “energy” $Q_{k}$ of the kth event is considered to be equal to the corresponding daily value of ∣ΔSLHF∣, provided that this is above a certain threshold ∣ΔSLHF∣${}_{thres}$. Then, the NT analysis is applied to the time series of the events of ∣ΔSLHF∣ as in the foreshock seismic activity.

**Figure 12:**
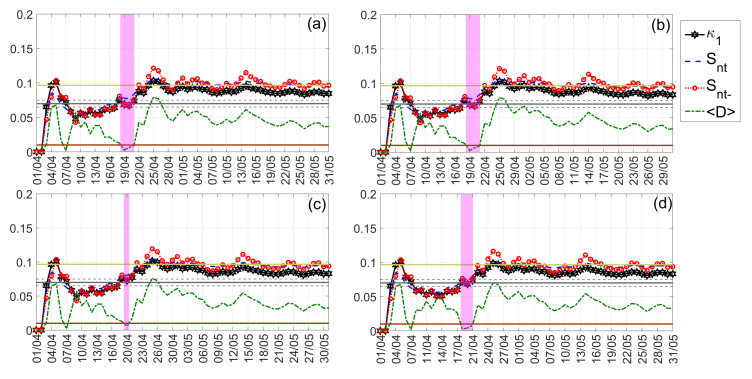
NT analysis of Nepal earthquakes ∣ΔSLHF∣ for the examined time period (1 April 2015 to 31 May 2015). The presented temporal variations in the NT parameters correspond to the different thresholds ∣ΔSLHF${|}_{thres}$: (**a**) 0, (**b**) 2, (**c**) 4, and (**d**) 6. The limit value of the entropy $S_{u}\left(\approx 0.0966\right)$ appears as a horizontal solid light green line, while the $\kappa_{1}$ value 0.07, along with a region of ±0.005 around it, is denoted by a horizontal solid gray and two horizontal dashed gray lines, respectively. The $10^{-2}$〈D〉 limit is shown as a horizontal brown line. The events presented in each panel depending on the corresponding threshold. In addition, although the conventional time (Date) of occurrence of each corresponding event is noted in x-axis tick values, the x-axis scale actually follows the NT representation; for this reason, the x-axis is not linear in conventional time.

**Figure 13:**
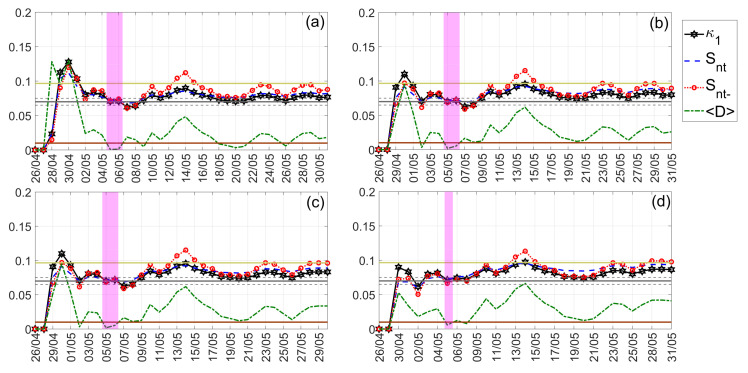
NT analysis of Nepal earthquakes ∣ΔSLHF∣ for the examined time period (26 April 2015 to 31 May 2015). The presented temporal variations of the NT parameters correspond to the different thresholds ∣ΔSLHF∣${}_{thres}$: (**a**) 0, (**b**) 2, (**c**) 8, and (**d**) 12. Figure format follows the format of Figure 12.

The NT analysis results for the case of Nepal earthquakes are illustrated in Figure 12. The ∣ΔSLHF∣ values analyzed correspond to the time period from 1 April 2015 to 31 May 2015. As one can see from Figure 12, all criticality conditions are satisfied for the date 20 April 2015, i.e., 5 days prior to the M7.8 Nepal earthquake (25 April 2015). Moreover, we should mention that the criticality condition demanding that the parameter $\kappa_{1}$ should approach the value $\kappa_{1}=0.07$ “by descending from above” could be said to be marginally satisfied, in the sense that this “descending from above” takes place during a relatively short time period and starting from values close to $\kappa_{1}=0.07$. It is also worth noting that, by analyzing the time period from 1 April 2015 to 31 May 2015, no approach to criticality was identified prior to the M7.3 Nepal earthquake of 12 May 2015. This is probably due to the fact that when including data embedding the critical dynamics related to the 25 April 2015 earthquake, these are “masking” any critical dynamics in the ∣ΔSLHF∣ time series that might be related to the following 12 May 2015 earthquake. To test this hypothesis, we also applied NT analysis to the same time series but starting after the occurrence of the M7.8 Nepal earthquake, i.e., for the time period 26 April 2015 to 31 May 2015. These results are presented in Figure 13, showing that, indeed, the ∣ΔSLHF∣ time series approaches criticality on 5 May 2015, i.e., 1 week before the M7.3 Nepal earthquake of 12 May 2015.

**Figure 14:**
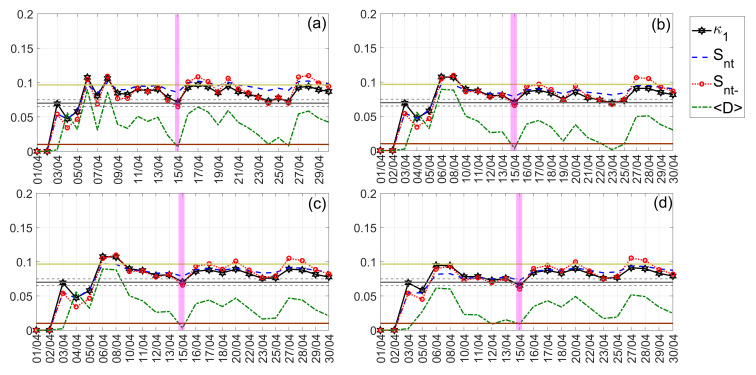
NT analysis of Kumamoto earthquakes ∣ΔSLHF∣ for the examined time period (1 April 2016 to 30 April 2016). The presented temporal variations in the NT parameters correspond to the different “low” thresholds ∣ΔSLHF∣${}_{thres}$: (**a**) 0, (**b**) 4, (**c**) 6, and (**d**) 8. Figure format follows the format of Figure 12.

**Figure 15:**
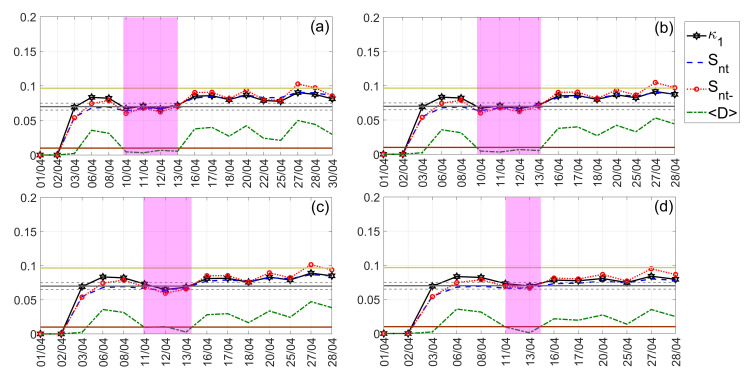
NT analysis of Kumamoto earthquakes ∣ΔSLHF∣ for the examined time period (1 April 2016 to 30 April 2016). The presented temporal variations in the NT parameters correspond to the different “high” thresholds ∣ΔSLHF∣${}_{thres}$: (**a**) 20, (**b**) 30, (**c**) 32, and (**d**) 40. Figure format follows the format of Figure 12.

The NT analysis results obtained for the Kumamoto earthquake are shown in Figure 14 and Figure 15 for “low” and “high” threshold values, respectively. Low thresholds (Figure 14) that take into account even less “significant” events, namely, lower ∣ΔSLHF∣ values, indicated that the underlying system approaches criticality on 15 April 2016, i.e., on the day of the M7.0 mainshock. However, when excluding less “significant” events from NT analysis, by considering higher ∣ΔSLHF∣${}_{thres}$ values, the critical state is approached 4–2 days before the M7.0 mainshock, i.e., on 11–13 April 2016, as shown in Figure 15.

At this point, we would like to mention that any critical dynamics detected could be the result of any complex process reflected in the specific observable. Beyond earthquake preparation, such processes could be the preparation of extreme meteorological phenomena that affect heat flux, such as typhoons or cyclones. In the case of the Kumamoto earthquake, a cyclone started developing on 14 April 2016 and reached a maximum intensity from 16 to 18 April 2016. Therefore, it is considered that the specific cyclone’s preparation is not expected to have influenced the NT analysis results for the daily-valued ΔSLHF(t). For the Nepal earthquakes case, there were no extreme meteorological phenomena at the nearest grid point during the studied time period.

One final test we conducted was to analyze the SLHF data from an absolutely “quiet” period, i.e., a time period without any earthquakes or other phenomena that could affect the dynamics of the SLHF. Although it was quite a demanding task to find such a quiet period of duration comparable to the above analyzed time periods, we managed to find that January 2019 (1–31 January) at the location of Nepal’s nearest grid point (see Table 1) was such a quiet period. The NT analysis of the corresponding SLHF data did not show an approach to criticality.

Especially for the Kumamoto earthquakes of 2016, it is interesting to briefly recall criticality analysis results that have recently been presented for various seismo-electromagnetic signals [42,51,52,53,54], as well as for the global navigation satellite system surface deformation [44] possibly related to Kumamoto earthquakes and the LAIC phenomenon. First of all, the 15 April 2016 M7.0 earthquake has been proven, by means of identified critical dynamics and evidence for departure from critical state in the ultra-low frequency (ULF) magnetic field fluctuations, to be the mainshock, rendering the M6.2 and M6.0 foreshocks that preceded it on 14 April 2016 [54]. Moreover, critical dynamics in the ULF magnetic field fluctuations have been found 5–1 days before the mainshock [42,54], as well as ∼4–2 weeks before the mainshock as resulted from the analysis of specifically defined daily valued ULF magnetic field quantities (∼2.5–2 weeks before for the $F_{h}$ lithospheric ULF radiation quantity, ∼4–3 weeks before for the ionospheric signature of the depression of the horizontal magnetic field component $Dep_{h}$, and ∼3 weeks before for $\delta Dep_{h}$) [42,51].

The analysis of very-low-frequency (VLF) sub-ionospheric propagation data received at multiple receivers has also revealed that the lower ionosphere reached criticality ∼1 week to 3 days before the mainshock as resulted from the analysis of the raw receiver data [42,52], while, when analyzing specifically defined daily valued VLF propagation quantities, the approach to criticality has been identified within the period of 10–2 days before the M7.0 mainshock [42,53]. Finally, it has been found that for the daily sampled GNSS deformation data and its fluctuations in two AGW bands of 20–100 and 100–300 min, approach to criticality in the period of 28 March to 14 April 2016 was found for different GNSS observing stations’ data. This evidence peaked on 28 March, 5 April, and 11–13 April, while on 11–14 April, 4–1 days before the mainshock, criticalities were frequently observed at all five studied stations [44]. These findings are likely to be consistent in time with the stratospheric result by Yang et al. [44] and Yang et al. [50].

## 5. Conclusions

Thermal anomaly is one of the most important channels of the LAIC mechanism because it directly deals with the immediate after-effects of seismic hazards over the surface and lowermost atmospheric altitudes. Outgoing thermal radiation from the earthquake epicenter and fault lines is considered to modulate the temperature, latent heat, and relative humidity of the surface to the lower tropospheric height. In this article, a space-based observation of such thermal excitation is examined by NOAA satellite outputs. The SLHF seems to be a potent parameter of the LAIC process since significant intensification has been observed during, prior to, and after strong earthquakes. We examined such thermal behavior for three earthquakes: two that took place in Kathmandu, Nepal (25 April and 12 May 2015), and one in Kumamoto, Japan (15 April 2016). In our previous publication [6], we have studied another thermal parameter OLRs from a similar NOAA satellite observation where the direction of such an enhanced OLR had a longitudinal extension (east–west). In contrast to the OLR, which is a directly emerging thermal wave in the infrared range, the SLHF anomaly is rather differently originated by the attachment of water molecules to thermal ions.

In this paper, it is the first time that natural time (NT) analysis is used to find the approach of the criticality of the SLHF before earthquakes. NT analysis is a physics-based method to analyze the time series of complex systems’ observables which is able to detect critical dynamics. Previously, the NT analysis method has been used on different electromagnetic parameters to identify the approach of criticality before earthquakes, e.g., [51,52,53,54]. In our study, we used NT analysis for the thermal channel of the LAIC mechanism. We found that the observed anomaly is in the same time range of the criticality approach. Our findings indicate that the atmosphere is in the critical stage before the studied earthquakes.

The criticality analysis, by means of the NT method, reveals that criticality was approached ≤1 week before each one of the studied Nepal earthquakes, specifically, 5 days before the 25 April 2015 main event and 1 week before the 12 May 2015 mainshock. Interestingly, in order to disclose the criticality dynamics related to the latter one, we had to run the criticality analysis by employing the SLHF data after the occurrence of the April 2015 event. In case all the available data (including dates even prior to the 25 April event) are used, a masking phenomenon takes place, where the critical dynamics related to the April event do not allow for the critical dynamics of the May event to be revealed. Moreover, on 15 April 2016, the Kumamoto mainshock criticality is approached 4–1 days before the earthquake. The data analysis indicates that the atmosphere was in the critical state before all the studied earthquakes.

As far as the direct pre-seismic SLHF anomalies evidence presented in this work are concerned, we have to mention that the anomalies revealed by the spatio-temporal variation analysis happened in close dates to the approach to criticality, within 1 week before each mainshock. One difference between the Nepal events and the Kumamoto event is that for the Nepal cases, criticality precedes the seismogenic spatio-temporal anomaly, while in the Kumamoto case, the opposite is observed.

Focusing on the 2016 Kumamoto earthquake, for which several statistical and criticality analyses of observables related to the LAIC process have recently been presented, we note that all the up to now studied observables (see Section 4) were found to embed critical dynamics during the last 10 days before the mainshock, with overlapping time durations. This clearly corroborates the LAIC hypothesis since these criticality analysis results suggest that the lithosphere, atmosphere, and ionosphere were indeed coupled and can be considered as a system, and this lithosphere–atmosphere–ionosphere system is at a critical state before the mainshock occurrence. However, due to the overlaps of the criticality time periods, it is difficult for one to extract detailed cause-and-effect information.

So far in our approach, we focused on this problem and succeeded in showing the changes as well as the reasons. There are so many significant parameters associated with the thermal channel of LAIC which describe the changes of the lithosphere as the initial agent of the LAIC process. However, the exact way of understanding the reason behind those changes remains unknown. The radon emission process over the earthquake epicenter has been found to be the initial threshold of the LAIC mechanism that excites the air ionization process. The newly formed ions coagulate with water molecules, known as the ion hydration process. In addition, the cluster of ions increase and the relative humidity decreases. These processes lead to the increase in the air temperature and change in air conductivity by generating the anomalous electric field. The change in air conductivity also increase the air temperature and modify the air pressure gradient. All these processes simultaneously change ionospheric potential, create vertical thermal convection, and generate atmospheric gravity waves. To understand the entire process of the LAIC mechanism, one has to study and take into account the all-possible parameters related to the entire process. Multi-parametric and multidisciplinary approaches will able to help us to understand the LAIC process starting from the lower atmosphere to upper ionosphere [55]. In parallel, other thermal parameters such as relative humidity, air temperature, surface temperature, cloud coverage, etc. can be utilized as a potent component of seismogenic thermal excitation. Continuous monitoring of such parameters is thus an absolute need, and we need to study those phenomena globally, as well. This will help us to grasp the entire scenario on how those physical processes are actually interrelated and propagate at the same time to produce the pre-seismic anomalies. Finally, further coordination of thermal anomalies with the information of ionospheric and magnetospheric perturbations as a multidisciplinary work will be extensively planned better to elucidate the LAIC process of our greatest concern. These will be studied in the future and will be published elsewhere. 

## Figures and Tables

**Table 1:** Nearest Grid Point Information.

Earthquake	Location of	Richter Scale	Depth (km)	Date and	Nearest Grid	Nearest Grid	Distance of Nearest
	Epicenter	Magnitude		Time (UT)	Point Latitude	Point Longitude	Grid from Epicenter (km)
Nepal	$27.837^{\circ }$ N,	7.3	18.5	12/05/2015	$27.6186^{\circ }$ N	$86.25^{\circ }$ E	29.66
	$86.077^{\circ }$ E			07:05:19			
Kumamoto	$32.782^{\circ }$ N,	7.0	10	15/04/2016	$33.3328^{\circ }$ N	$131.25^{\circ }$ E	78.33
	$130.726^{\circ }$ E			16:25:06			

## Data Availability

The data are freely available from the NOAA Reanalysis repository https://psl.noaa.gov/data/gridded/data.ncep.reanalysis.html (accessed on 23 September 2018). From this repository, we use the Surface fluxes daily data in our study.
